# Micronutrient deficiencies in African soils and the human nutritional nexus: opportunities with staple crops

**DOI:** 10.1007/s10653-019-00499-w

**Published:** 2020-01-04

**Authors:** J. Kihara, P. Bolo, M. Kinyua, J. Rurinda, K. Piikki

**Affiliations:** 1 International Center for Tropical Agriculture (CIAT), Nairobi, Kenya; 2 International Plant Nutrition Institute (IPNI), Nairobi, Kenya; 3 Swedish University of Agricultural Sciences (SLU), Skara, Uppsala, Sweden

**Keywords:** Micronutrients, Fertilization, Sub-Saharan Africa, Human nutrition, Soil fertility management, Biofortification, Profitability

## Abstract

A synthesis of available agronomic datasets and peer-reviewed scientific literature was conducted to: (1) assess the status of micronutrients in sub-Saharan Africa (SSA) arable soils, (2) improve the understanding of the relations between soil quality/management and crop nutritional quality and (3) evaluate the potential profitability of application of secondary and micronutrients to key food crops in SSA, namely maize (*Zea mays* L.), beans (*Phaseolus* spp. and *Vicia faba* L.), wheat (*Triticum aestivum* L.) and rice (*Oryza sativa* L.). We found that there is evidence of widespread but varying micronutrient deficiencies in SSA arable soils and that simultaneous deficiencies of multiple elements (co-occurrence) are prevalent. Zinc (Zn) predominates the list of micronutrients that are deficient in SSA arable soils. Boron (B), iron (Fe), molybdenum (Mo) and copper (Cu) deficiencies are also common. Micronutrient fertilization/agronomic biofortification increases micronutrient concentrations in edible plant organs, and it was profitable to apply fertilizers containing micronutrient elements in 60–80% of the cases. However, both the plant nutritional quality and profit had large variations. Possible causes of this variation may be differences in crop species and cultivars, fertilizer type and application methods, climate and initial soil conditions, and soil chemistry effects on nutrient availability for crop uptake. Therefore, micronutrient use efficiency can be improved by adapting the rates and types of fertilizers to site-specific soil and management conditions. To make region-wide nutritional changes using agronomic biofortification, major policy interventions are needed.

## Introduction

Soil nutrients status and management not only determine crop productivity but nutrients concentration in plant parts consumed as food and feed. Consequently, soil nutrients status has great implications on human health. At global scale, about one-third of arable soils are deficient in micronutrients, particularly in zinc (Zn) (Cakmak et al. 2017), and this eventually affects human nutrition. Approximately 2–3 billion people worldwide are suffering from micronutrient deficiencies, especially in developing countries where these affect at least half of the population (Goudia and Hash 2015). The problem of micronutrient deficiencies in soils (often involving 2–5 micronutrients at a time) is particularly widespread in SSA (Berkhout et al. 2017; Hengl et al. 2017). This is mainly a result of decades of soil degradation and low and unbalanced application of fertilizers mostly of nitrogen (N), phosphorous (P) and potassium (K). The importance of micronutrients in crop productivity was recently demonstrated (Kihara et al. 2017), but a huge gap remains in understanding their links to nutrition.

The nutritional quality of crop produce influences human nutrition either directly or indirectly (Dimkpa and Bindraban 2016). Consumption of food crops deficient in micronutrients (due partly to lack of adequate micronutrients in the soil, Manzeke et al. 2019) could occasion deficiency of such micronutrients in humans, often referred to as “hidden hunger” (Joy et al. 2015). Hidden hunger, the challenge widely documented in the 2014 Global Hunger Index report (von Grebmer et al. 2014), is largely a problem of inadequate intake of micronutrients. The severity of this in SSA has been demonstrated through ratings of the global hunger index, which is based on undernourishment, child underweight and child mortality. With the exception of Ghana and Gabon, most countries in SSA have global hunger index ratings of between serious and extremely alarming (von Grebmer et al. 2014). Agronomic biofortification through micronutrient application to crops (in soil or foliar) has the potential to ameliorate micronutrient deficiencies and improve crop productivity and nutritional quality of produce.

Food consumption patterns in Africa, especially among resource-constrained small-holder farmers, are dominated by staple cereals including maize and rice. However, micronutrients (especially Zn) deficiency in humans is mostly common in areas where cereals grown in micronutrient-deficient soils dominate the diets (Zou et al. 2012; Dimkpa and Bindraban 2016). Globally, Zn and iron (Fe) predominate the list of micronutrients commonly limiting in human diets (White and Broadley 2009; Stein 2010). For instance, although a concentration ranging between 40 and 60 mg Zn kg^−1^ in maize grain is recommended for human consumption (Pfeiffer and McClafferty 2007), less than 35 mg Zn kg^−1^ was contained in maize grain produced in the Zn-deficient soils in Zimbabwe (Manzeke et al. 2012, 2019).

The extent to which agronomic management practices enhance changes in crop nutritional quality, due especially to micronutrients, is hardly studied in SSA. For a long time, many studies in SSA have been focusing on the impact of macronutrients on crop productivity (e.g., Kihara et al. 2017). Of the few studies that have assessed the impact of Zn fertilizers on Zn concentrations in grains of major food crops, only two have focused on Africa, (i.e., a study conducted in Zambia by Zou et al. (2012) and in Zimbabwe by Manzeke et al. (2014). Nevertheless, interest has now been growing and only a few months ago a comprehensive study on mapping soil nutrient status of Zn and Fe across 350 locations in two agro-ecological regions of Zimbabwe was published (Manzeke et al. 2019). There are also studies that have now included crop quality data such as the recent soil nutrient diagnostic trials of Africa Soil Information Service (AfSIS), the Taking Maize Agronomy to Scale in Africa (TAMASA) and the Optimizing Fertilizer Recommendations in Africa (OFRA) projects) that provide an opportunity for wide-scale assessments to complement the scanty data in peer-reviewed publications.

Evidence showing that agricultural interventions are profitable is important to derive their adoption by partners and farmers. While the assessment of profitability of agronomic interventions has been undertaken for macronutrients (e.g., Kihara et al. 2016), no information on profitability of secondary and micronutrient fertilization on yields and nutritional quality of crops is available for SSA. Recently, positive changes in crop yields have, however, been observed when micronutrient fertilizer was applied in Africa (Kihara et al. 2017), and this provides a basis for a comprehensive assessment of the profitability of micronutrient fertilization. Elsewhere, for example in India, Dar (2004) showed that application of boron (B) and sulfur (S) was profitable for both soybean and wheat. The objectives of this study were:

1. To assess the extent of micronutrient deficiencies in SSA arable soils based on soil analysis, crop grain and quality response datasets,
2. To improve the understanding of the relations between soil quality/management and food crops’ nutritional quality and thereby provide the potential estimates of nutritional benefits of agronomic biofortification, by application of micronutrient fertilizers,
3. To evaluate the profitability of application of secondary and micronutrients in production of key food crops in SSA.

This study focuses on the SSA region where malnutrition and hidden hunger are key problems, and the extent of soil micronutrient deficiencies is extensive.

## Methods

### Extent of micronutrient deficiency in SSA

We evaluated the extent of micronutrient deficiencies in arable soils of SSA based on literature review focusing on data and reports of: (1) soil test-based assessments, for example, by Hengl et al. (2017) and Berkhout et al. (2017), (2) crop grain yield responses to nutrients, for example, by Kihara et al. (2017) and (3) micronutrient element concentrations in plant parts (grain, stover and leaves of the different crops).

### Soil and crop data acquisition

**Fig. 1:**
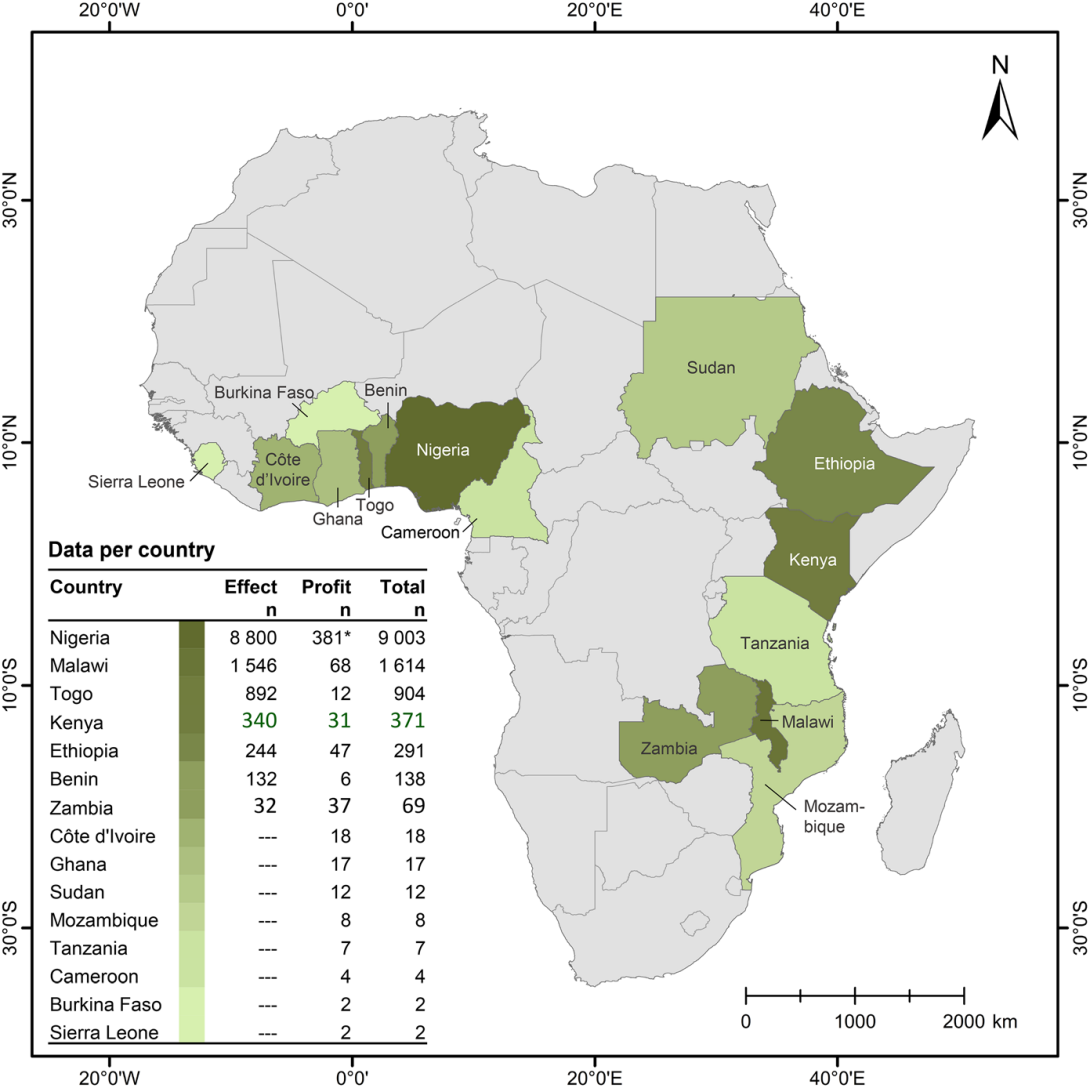
Data entries (*n*) obtained and used in the analysis of quality effects and profitability of micronutrients in sub-Saharan Africa covering seven crops: maize (*Zea mays* L.), wheat (*Triticum aestivum* L.), rice (*Oryza sativa* L.), cowpea (*Vigna unguiculata* L.), pearl millet (*Pennisetum glaucum*), finger millet (*Eleusine coracana Gaertn*.) and sorghum (*Sorghum bicolor* L.) and selected micronutrient elements (not all combinations present). * = 178 of these were also used for analysis of effects. Background map: Food and Agriculture Organization of the United Nations. FAO GEONETWORK. Global Administrative Unit Layers (GAUL) (GeoLayer). (Latest update: 04 Jun 2015)

The agronomic data used in this study were derived from multiple sources. First, we used a database on assessment of crop response to secondary and micronutrients in SSA by Kihara et al. (2017). This database was refreshed by additional literature searches. Additional data published by Manzeke et al. (2019) and by Wortmann et al. (2019a) were then included. To extend the dataset, unpublished plant analyses data containing micronutrient concentrations (in grain, stover and ear leaves) and grain yields were obtained from different sources (Table 1; Fig. 1). This included AfSIS data from Malawi and Kenya, Africa RISING data from Ethiopia, TAMASA data from Nigeria (Shehu et al. 2018), data obtained from specific researchers, e.g., omissions trials data conducted in Nigeria and Togo (Nziguheba et al. 2009). All these datasets contain plant nutrient concentrations such as calcium (Ca), magnesium (Mg), S, manganese (Mn), B, molybdenum (Mo), Fe and copper (Cu), besides Zn, which is the most commonly reported plant micronutrient in SSA.

**Table 1:** Summary of plant micronutrient concentrations data obtained from different sources in sub-Saharan Africa, for the crops, maize (*Zea mays* L.), wheat (*Triticum aestivum* L.), rice (*Oryza sativa* L.), cowpea (*Vigna unguiculata* L.), pearl millet (*Pennisetum glaucum*), finger millet (*Eleusine coracana* Gaertn.) and sorghum (*Sorghum bicolor* L.)

Country	Crops	Crop parts	Micronutrients	Count	Data sources
Benin	Maize	Leaves	Zn	100	Diagnostic trials
Ethiopia	Wheat	Grain	S	24	Habtegebriel and Singh (2009)
Ghana	Cowpea, maize	Shoots	Zn	84	Wortmann et al. (2019a, b)
Kenya	Maize	Leaves	Zn	1014	AFSIS
Malawi	Cowpea, maize	Grain, leaves	Zn, Se, Cu, Mn, S,	2570	Chilimba et al. (2012/2014), Wortmann et al. (2019a, b), AFSIS
Mali	Maize, pearl millet	Shoots	Zn	333	Wortmann et al. (2019a, b)
Niger	Cowpea, maize, pearl millet, sorghum	Shoots	Zn	1232	Wortmann et al. (2019a, b)
Nigeria	Maize, sorghum	Shoots, leaves	Zn, Mn, S, B	6246	AFSIS, TAMASA, Nziguheba et al. (2009), Wortmann et al. (2019a, b)
Rwanda	Maize, sorghum	Shoots	Zn	28	Wortmann et al. (2019a, b)
Tanzania	Cowpea, maize, sorghum	Grains, shoots	Zn, Cu	394	Wortmann et al. (2019a, b), Lisuma et al. (2006)
Togo	Maize	Leaves	Zn, Mn, S, B	1115	Diagnostic trials
Uganda	Finger millet	Shoots	Zn	665	Wortmann et al. (2019a, b)
Zambia	Maize, wheat	Grains, shoots	Zn	117	Zou et al. (2012); Ram et al. (2015), Wortmann et al. (2019a, b)
Zimbabwe	Cowpea, finger millet, maize, sorghum	Grain	Zn	706	Manzeke et al. (2019)

*AfSIS* = Africa Soil Information Service; *TAMASA* = Taking Maize Agronomy to Scale in Africa

Price data for different micronutrient fertilizers were obtained from both local and international fertilizer suppliers. Unlike macronutrients, there was no online site or portal offering micronutrient fertilizer prices, so personal contacts to the major fertilizer suppliers were made to acquire up to date prices (Table 2). Since the majority of the price information for the inputs (micronutrient fertilizers) and outputs (maize yields) were mostly from sources in Kenya, there might be slight variations in prices across SSA.

**Table 2:** Prices (as of May, 2019) and sources of micronutrients used in the study

Fertilizer compound	Target element	Proportion element (%)	Price of element (US$ kg^−1^)	Source
Zinc chelate (EDTA)	Zn	15.03	16.2	Global green planet
Zinc sulfate	Zn	40.5	4.3	Ocean agriculture
Ammonium sulfate	S	24.27	2.3	Ocean agriculture
Iron sulfate	Fe	36.76	6.0	Skylab Nairobi
Sodium pentaborate	B	3.66	6.2	Hemal impex (Indiamart.com)
Copper sulfate	Cu	39.8	14.6	Ocean agriculture

In calculations of the micronutrient element price, the amount (percentage) of the micronutrient element in the different fertilizer products was first calculated based on the molecular masses of the constituents in the compound fertilizer. The price of the compound fertilizer product was divided by the total amount (percentage) of the target micronutrient element contained to obtain price in kilogram of the micronutrient element. The total price of the target micronutrient was finally arrived at by multiplying the resultant cost per kilogram with quantity of secondary or micronutrient element applied per hectare. Where the treatment involved two or more of the secondary and micronutrients, i.e., “combined,” the total cost for the different fertilizer products used was considered.

Variable sources of secondary and micronutrient fertilizers were used in the compiled studies. These sources included: zinc sulfate, zinc sulfate monohydrate, zinc chelate, zinc oxide, zinc carbonate and zinc chloride for Zn; sodium borate, boron chloride and sodium pentaborate for B; sodium molybdate for Mo; copper sulfate and copper oxide for Cu; ammonium sulfate, potassium sulfate, sodium sulfate, ammonium sulfate nitrate, single superphosphate and gypsum for S; iron sulfate for Fe and sodium selenite for Se. Some of these fertilizer sources are used for their availability on the market, but not because of being the cheapest sources of the required secondary and micronutrients. Unless in chelated form with high nutrient concentration of the products, the cheapest sources of the nutrients were used in the present analyses. Our estimates are conservative as they assume that the fertilizer product is bought solely for supplying a particular secondary or micronutrient of interest. For S from ammonium sulfate (still the cheapest way to acquire sulfur), for example, the price would reduce from US$ 2.3 kg^−1^ (Table 2) to about US$ 1.8 kg^−1^ if the known price of the contained nitrogen is subtracted.

Cost of transport of micronutrients was not taken into account as this is easily absorbed in transport costs of macronutrients. (Farmers normally buy micronutrients together with macronutrients.) However, when micronutrient products are not available, farmers would travel beyond common distances of between 2 and 5 km to agro-dealer outlets (McCall 1985; Misiko 2012). Our analyses assume a scenario where the products are available locally.

Net benefits were calculated as the difference between additional gross income and costs of the secondary and micronutrient fertilizers used. Gross income was obtained by multiplying micronutrient yield response, calculated by subtracting the control yield from the micronutrient yield, with the output prices. These agricultural outputs (yield) prices were averaged for 5 years ranging from January 2014 to January 2019 obtained from FAO (http://www.fao.org/giews/food-prices accessed on January 29, 2019). All the prices were for countries in Eastern Africa, i.e., rice (*Oryza spp.)* for Tanzania, wheat (*Triticum aestivum* L.) and sorghum (*Sorghum bicolor* L.) for Ethiopia and maize (*Zea mays* L.) for Kenya, representing the dominant crops in each of these countries. Soybean (*Glycine max* L.) and cowpea (*Vigna unguiculata* L.) prices were not available in the FAO portal and were obtained from local outlets in Kenya, e.g., National Farmers Information System (nafis.go.ke) and kilimo.go.ke.

### Data analysis

#### Soil and plant micronutrient analysis

Boxplots showing distributions of concentrations of specific nutrients in harvested plant parts for different crops were generated using R statistical program. The boxplots show the median of the data, the interquartile range represented by the middle “box” and distributions of data beyond the lower and upper quartiles. To show critical thresholds as reference in interpreting the nutrient concentrations, broken lines indicating minimum critical values were added to the plots.

The means of maize ear leaf Zn, Mn, B and S concentrations for selected treatments (control [no input], NPK and NPK plus secondary and micronutrients) were obtained from the raw data. For this, bootstrap confidence limits were obtained using boot R package with 100 replications.

For Zn, co-located data for both plant and soil tests were available allowing us to show the overall distributions of the concentrations, but also how these are influenced by the available levels in the soils. For this, scatter plots of plant Zn concentrations (in grains and leaves) for different crops against soil test Zn concentrations were undertaken in Microsoft Excel.

#### Economic analysis of micronutrients use

Profitability analysis of micronutrient application using the available experimental data in SSA was undertaken with caution. Agronomic trials of crop responses to micronutrients, especially where factorial designs are used, often apply exclusion principles that demand a fertilizer product that may come at a very high price just to eliminate another nutrient of interest to a research project. For example, to avoid nitrogen in –N+S treatments, Habtegebrial and Singh (2009) used potassium sulfate that is more expensive than ammonium sulfate used in the +N+S treatments. The source of micronutrient was not provided in Chaguala et al. (2011) and was assumed to be the most commonly used, being ammonium sulfate for S (the treatment needed N as well) and zinc sulfate for Zn. The calculated micronutrient prices could be slightly over-estimated, since only the target micronutrient was costed from the compound fertilizer.

Cumulative frequency distributions of net benefits for the individual crops and nutrient applications were derived when the number of observations was at least 30. The distributions were plotted in R statistics software with the cumulative probability represented by a graph of the cumulative distribution function. This displays the benefits, sorted in increasing order, and their whole range is shown in the x-axis.

## Results and discussion

### Extent of micronutrient deficiencies in sub-Saharan Africa

The available micronutrient contents in arable soils for much of SSA are below critical thresholds (Toenniessen et al. 2008). Previous review on micronutrient problems in west Africa pointed to boron, zinc and molybdenum deficiencies as the most prevalent (Abe et al. 2010; Buri et al. 2000), while the highest deficiencies for boron and copper occur in the sub-humid zones (Hengl et al. 2017). Based on their review, Kihara et al. (2017) pointed out major hotspots for widespread deficiencies in micronutrients in Ivory Coast, Nigeria, Togo, Democratic Republic of Congo, Kenya, Sudan, Ethiopia, Ghana, Malawi, Sierra Leone, Tanzania, Zambia as well as Burkina Faso. Other researchers have reported widespread deficiencies, e.g., for Zn, S and B in Ethiopia (Vanlauwe et al. 2015) and S in Malawi (Chilimba and Chirwa 2000). Although deficiencies of secondary and micronutrients are associated with continued mining by crops because of non-application of these nutrients in production, there is no long-term data in SSA to show their trends in the soil.

In some regions of SSA, multiple deficiencies of up to five micronutrients are prevalent (Berkhout et al. 2017). The most affected regions with the co-occurrence of micronutrient deficiencies (with up to five nutrients) are in the northern edge of the Sahel of West Africa, areas around the Congo basin, Eastern Africa, and Southern Africa (Berkhout et al. 2017). Due to agronomic associations, the deficiency of even one micronutrient can affect crop productivity and nutritional quality.

### Micronutrients and crop nutritional quality

The SSA region’s human nutritional requirements are fast soaring up, with its population estimated to hit 2.5 billion by the year 2050 (UN, DESA 2015). When not taken in adequate quantities, the essential micronutrients impact productivity and human health in a wide range of ways (Welch and Graham 2012).

#### Selenium (Se) in harvest grains

**Fig. 2:**
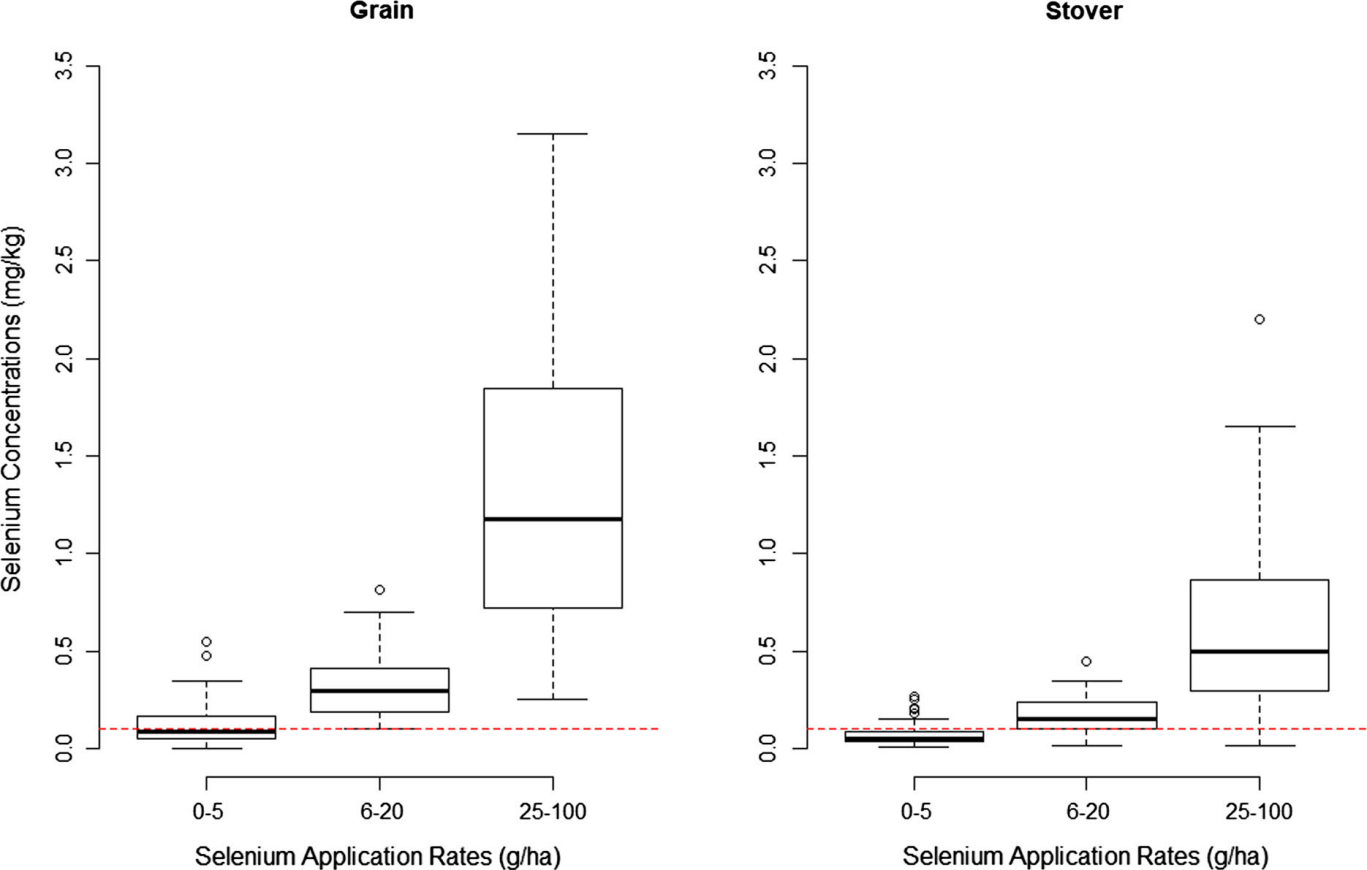
Selenium concentrations in maize grain and stover under different ranges of Se application rates in maize (*Zea mays* L.). The mid-line is the median. The box indicates interquartile range, while the whiskers show non-outlier range. The red lines show the lower critical limit of Se for humans

Crop nutritional quality was found to be improved through micronutrient applications. Selenium (Se), applied either as selenate or as selenite, is not required for plant growth, but it is a critical nutrient for animals and human beings. Topdressing pastures with Se at a rate of 10 g ha^−1^ prevents Se deficiency in livestock (Curtin et al. 2006). In Malawi, application of one gram of Se per hectare (from either Na_2_SeO_4(aq)_, NPK + Se or CAN + Se) increased maize stover quality and grain Se concentration by 15–21 μg Se kg^−1^ (Fig. 2; Chilimba et al. 2012).

In a follow-up study, application of 10 g Se ha^−1^ increased selenium grain concentration by eightfold (from 13 to 113 μg Se kg^−1^ grain) in maize, ninefold in groundnut (from 43 to 415 μg Se kg^−1^ grain) and 18-fold in soybean (from 45 to 813 μg Se kg^−1^ grain; Chilimba et al. 2014). Such increases are also observed elsewhere; in Australia, Se application (ranging from 4 to 120 g Se ha^−1^), either as soil or as foliar application, resulted in 133-fold and 20-fold increases in wheat grain Se concentrations, respectively (Lyons et al. 2003). Through such agronomic biofortification with Se, daily per capita maize-based Se intake can be increased to contribute a greater proportion of the recommended daily per capita Se intake of 50–70 μg (Chilimba et al. 2011).

From the present analysis, applying 1–5 g Se ha^−1^ is inadequate to attain the minimum acceptable range of Se concentrations in grains (0.1 mg Se kg^−1^ (Curtin et al. 2006); Fig. 2). Applying at least 6 g ha^−1^ Se results in selenium concentrations in grains above this minimum threshold. Even application of 25–100 g ha^−1^ Se still results in concentrations below 4 mg Se kg^−1^, the upper limit beyond which selenium toxicity can result in humans and livestock through food and feed (Underwood and Suttle 1999; Underwood 1977). In general, Se application has a positive effect on the nutritional value, and there is a very wide variation in crop response to its application, indicating the need for site-specific application rates.

The normal range of total Se in soils is given as 0.01–2.0 mg kg^−1^ (Saha et al. 2017). The available data for soil Se in Malawi, despite varying widely (over 12-fold), are still low, range of 0.05–0.62 mg Se kg^−1^ for total Se and 0.001 and 0.016 mg Se kg^−1^ for KH_2_PO_4_-extractable soil Se (Chilimba et al. 2011). Depending on the total selenium levels in the soil to meet the human Se nutrition demands, soils have been categorized as deficient (< 0.125 mg Se kg^−1^), marginal (0.125–0.175 mg Se kg^−1^), moderate to high (0.175–3 mg Se kg^−1^) and excessive (> 3 mg Se kg^−1^; Saha et al. 2017). Soils containing more than 5 mg Se kg^−1^ produce vegetation with Se in toxic levels for animal consumption. In Malawi, Chilimba et al. (2011) observed maize grain Se concentration that was up to tenfold higher for soils with high pH (> 6.5), and this is explained by greater Se availability to plants following greater solubility of selenium (Se(IV)) species and oxidation to selenate (Se(VI)). They also reported positive correlation between grain Se concentration and soil pH, especially at the high pH.

#### Copper (Cu) in harvest grains

**Fig. 3:**
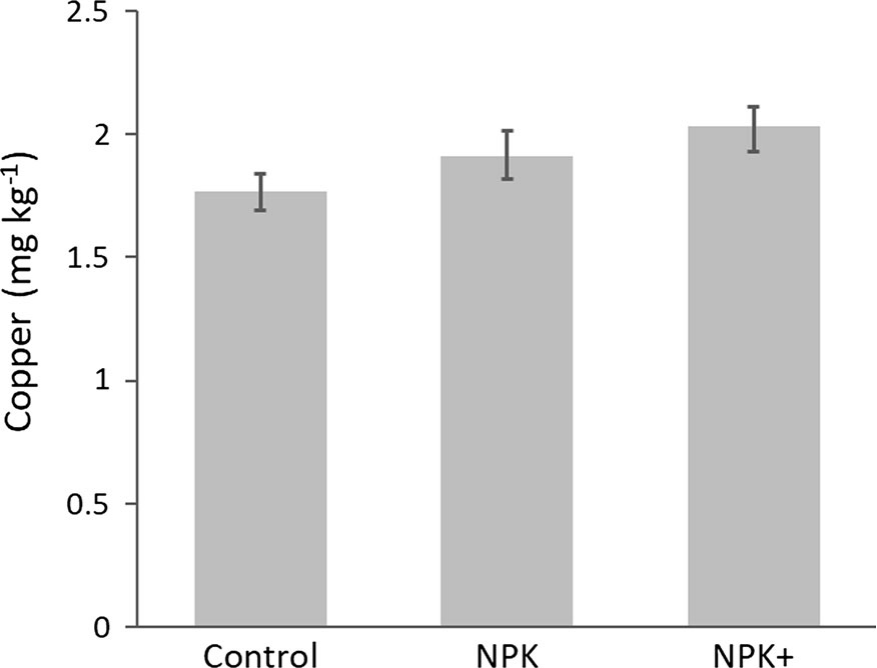
Effects of NPK fertilizer, and secondary and micronutrients on concentration of copper in maize grain (*Zea mays* L.) as observed in sub-Saharan Africa (Nigeria and Malawi). Error bars are bootstrap confidence intervals of means. Number of observations varied from 153/156 among the treatments. Control = no fertilizer added, NPK = fertilizer containing nitrogen (N), phosphorous (P) and potassium (K), NPK+ = fertilizer containing NPK and one or more micronutrient elements

Compared with the grain quality of unfertilized maize, the concentration of Cu increased in maize grain when NPK fertilizer plus secondary and micronutrients (including Cu) were applied (Fig. 3). Application of macronutrients alone did not increase significantly the concentration of Cu in maize grain over that of the control treatment (i.e., confidence limits of means for these treatments are overlapping) (Fig. 3).

#### Zinc in harvest grains

**Fig. 4:**
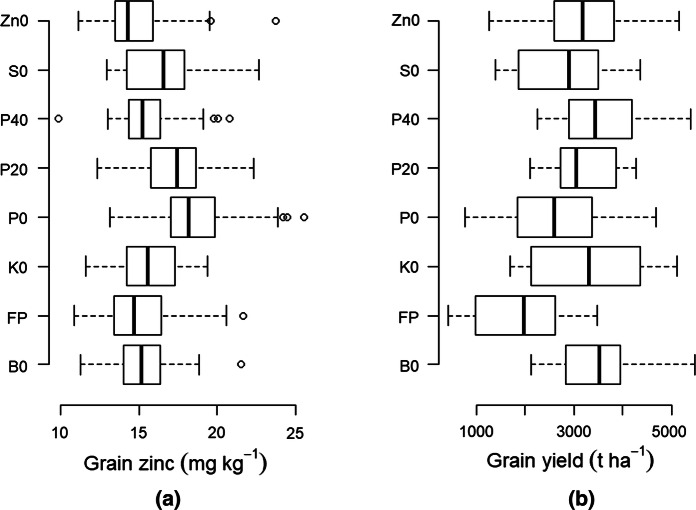
Boxplots showing zinc (Zn) concentration in maize (*Zea mays* L.) grain and the associated yields following nutrient omissions. Nutrient followed by zero means that the nutrient was omitted. FP = farmer practice (not fertilized)

**Fig. 5:**
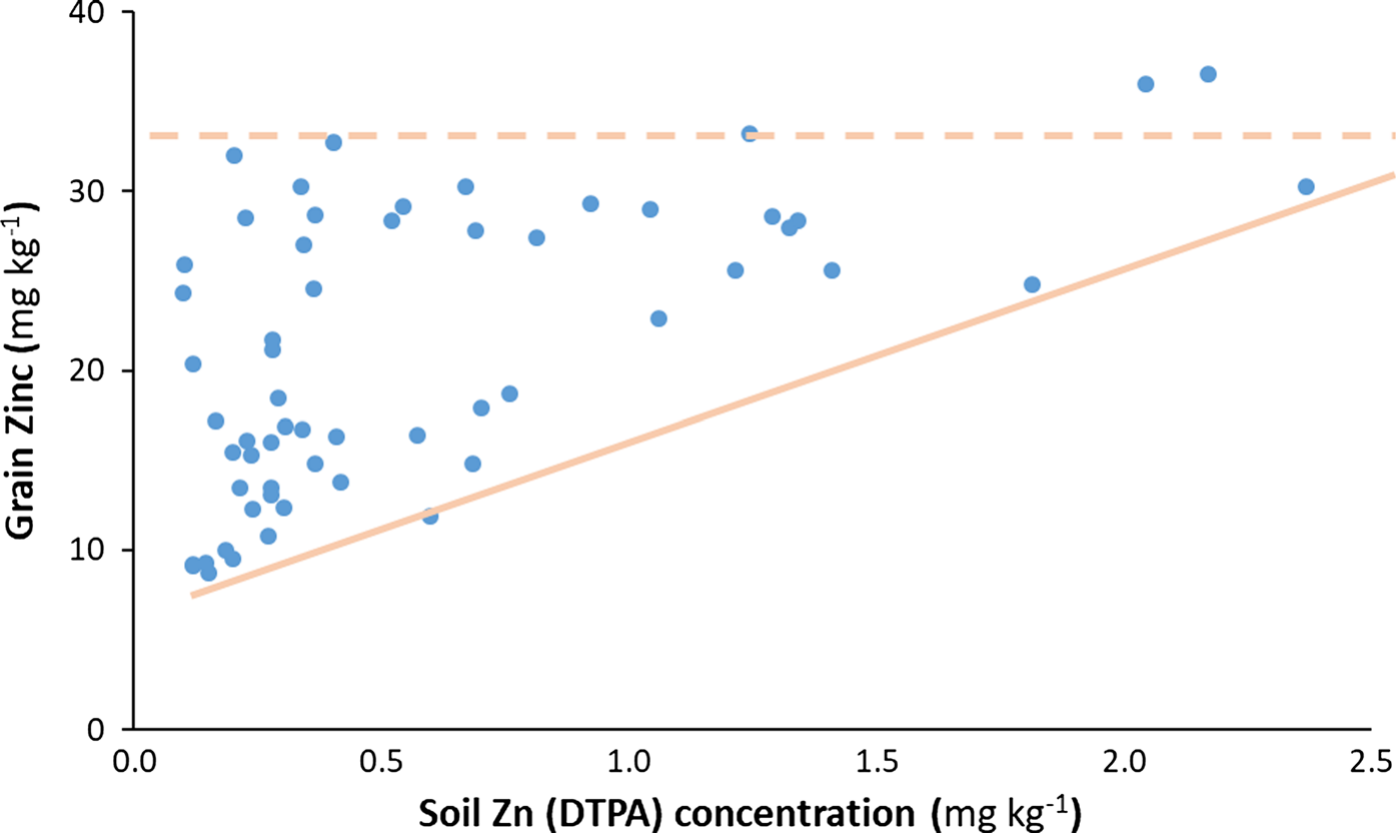
Grain zinc (Zn) concentrations in maize (*Zea mays* L.) at different soil Zn test values for different farms in Zimbabwe. Each data point represents an individual farm. Broken line indicates the similar maximum grain Zn concentrations, while the continuous line indicates trend for the lowest concentrations

Omission of Zn (farmer practice, (FP) and zinc omission, Zn0 treatments) has the lowest grain Zn concentrations (Fig. 4). Application of Zn without P (P0) or with low amounts of P (P20) resulted in the highest grain Zn concentrations. Due to the interactions between P and Zn in the soil, the uptake of Zn by plants decreased as the amount of plant available P increased. Unfortunately, all the observed maize grain Zn concentrations (Fig. 4) are below the global average of 25 mg Zn kg^−1^ (Ortiz-Monasterio et al. 2007). Even in southern Africa (Zimbabwe), maize grain Zn concentrations are still below the concentration target of 38 mg Zn kg^−1^ needed to meet human dietary needs by HarvestPlus (Bouis and Welch 2010; Fig. 5). The conclusion by Manzeke et al. (2019) that grain Zn (and Fe) in food crops in southern Africa is insufficient for adequate human nutrition applies also for other parts of sub-Sahara Africa. An interesting result is that while the maximum grain Zn is about 33 mg kg^−1^ regardless of soil Zn concentrations, the minimum grain Zn increases with soil Zn. Generally, Manzeke et al. (2019) observed that higher extractable soil Zn concentration was correlated with a higher grain Zn concentration in staple crops, with the extractable soil Zn influenced by management, soil organic matter content, total soil Zn and pH.

Studies involving Zn are more common than studies of other nutrients. Evidence has been building that Zn application, especially as foliar spray, increases wheat grain zinc concentrations, e.g., in Zambia (Zou et al. 2012) and Egypt (El-Dahshouri 2018) and maize grain quality in Egypt (Salem and El-Gizawy 2012) and Togo (Nziguheba et al. 2009; Fig. 4). According to Manzeke et al. (2014), Zn-based treatments increased grain Zn concentrations by 67% compared to treatments without Zn application. Joy et al. (2015) showed improvements of maize, rice and wheat grain zinc concentrations of 23, 7 and 19%, respectively, following soil Zn application and up to 30, 25 and 63% following Zn foliar application. In Nigeria, application of Zn at different levels resulted in Zn concentrations in maize shoots ranging between 3.15 and 10.40 mg kg^−1^, ranges that are still below the critical levels of 25–60 mg kg^−1^ Zn (Eteng et al. 2014). Moreover, application of Zn increased the quality of faba beans (*Vicia faba ssp*) through enhancing Zn concentrations in the nodule and upper matured leaves by 0.76 and 31 mg kg^−1^, respectively, in Ethiopia (Desta et al. 2015) relative to no Zn application. The increasing Zn concentration is sometimes associated with increase in grain protein (El-Dahshouri 2018; Seadh et al. 2009; El-Habbasha et al. 2015), pointing to the important functions of zinc in protein synthesis in the crop. In Turkey, Cakmak et al. (2010) observed that foliar application of Zn during early stage of seed development maximizes the accumulation of Zn in grain due to a possible high sink activity for Zn at this developmental stage. Crop growth stage, proper timing, splitting/frequency of application and concentrations, for example, of foliar zinc applications, are important factors influencing effects of micronutrient fertilization (Zhang et al. 2010; Boonchuay et al. 2013; El-Dahshouri 2018). Foliar zinc application between panicle initiation of paddy rice and 2 weeks after flowering resulted in higher grain zinc concentration than earlier application (panicle initiation and booting; Boonchuay et al. 2013) indicating more zinc reallocation to seed. Similar results are observed for wheat following foliar zinc application after flowering stage compared to before flowering stage (Ozturk et al. 2006; Cakmak et al. 2010; Phattarakul et al. 2012; Li et al. 2014), while Cakmak et al. (2010) obtained highest grain zinc concentrations when zinc was applied at four different growth stages (stem elongation, booting, early milk/dough and anthesis) compared to either two or three growth stages.

The application rates of Zn fertilizers depends on factors such as the soil chemical and physical properties, form of Zn fertilizer used, application method and crop species/cultivar. The critical level of Zn in soil below which deficiency occurs ranges from 0.6 to 2.0 mg Zn kg^−1^ depending on the Zn extraction method used (Singh et al. 2005). Application of Zn in the soil is often higher for zinc sulfate (2.5–22 kg Zn ha^−1^; Kinaci and Kinaci 2005; Sadeghzadeh 2013) than for chelated forms (0.3–6 kg Zn ha^−1^; Sadeghzadeh 2013). Depending on the Zn levels, plant Zn deficiency status can be categorized as definite Zn deficient (< 10 mg kg^−1^), likely to be deficient (10–15 mg kg^−1^), likely to be sufficient (15–20 mg kg^−1^) and sufficient (> 20 mg kg^−1^ of dry matter; Singh et al. 2005).

#### Sulfur and micronutrients in plant leaves

**Fig. 6:**
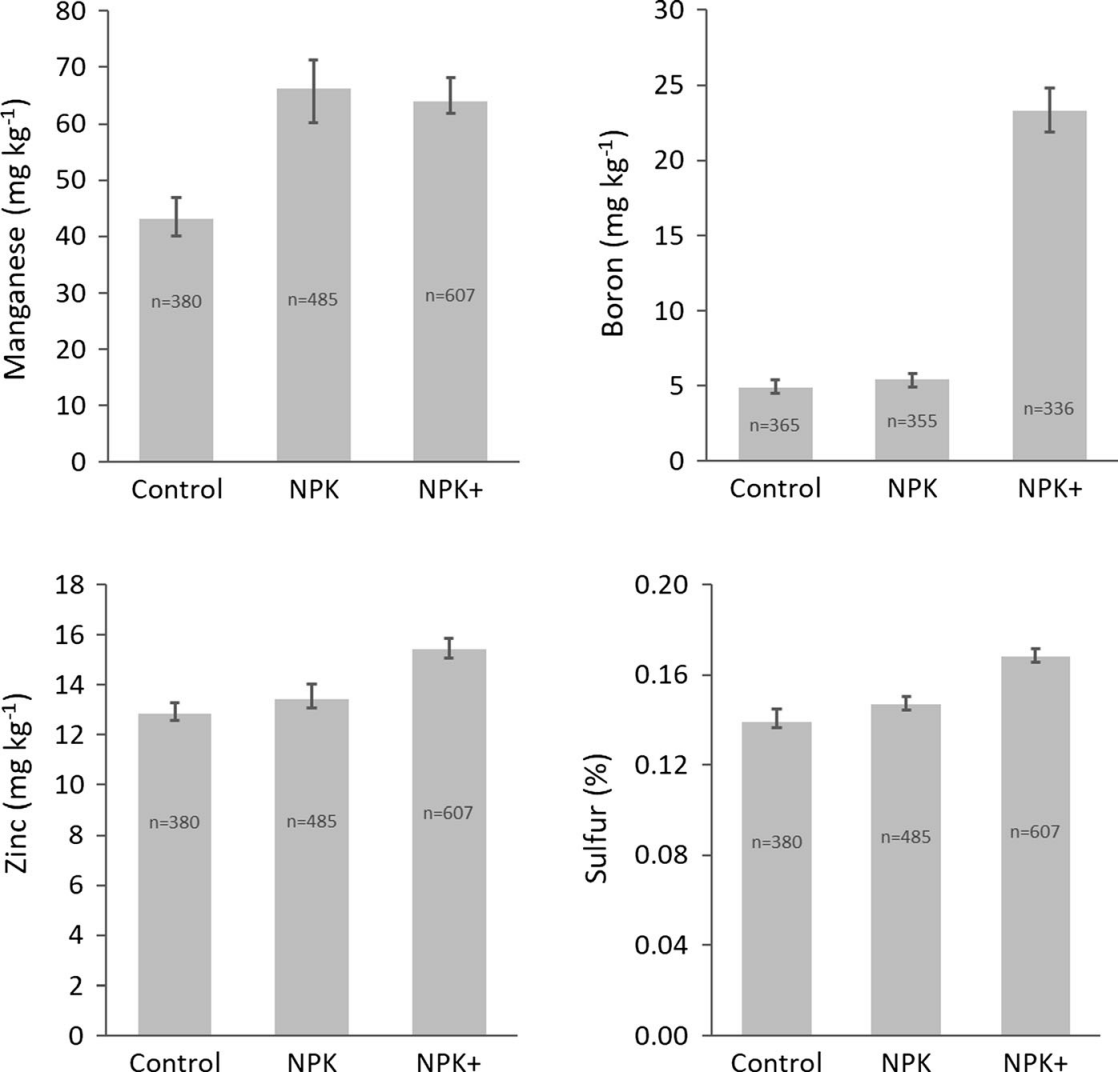
Effects of fertilizers including secondary and micronutrients on quality of ear leaves of maize (*Zea mays* L.) as observed in sub-Saharan Africa (Nigeria and Togo). Error bars show bootstrap confidence intervals of means. Control = no fertilizer added, NPK = fertilizer containing nitrogen (N), phosphorous (P) and potassium (K), NPK+ = fertilizer containing NPK (and one or more micronutrient elements)

Nutrient element concentrations in plant leaves can be good indicators of crop uptake and grain concentrations. Significant effects of fertilizer application on micronutrients are observed on ear leaves of maize. Concentrations of Mn in ear leaves of maize are enhanced by fertilizer application both with and without secondary and micronutrients (Fig. 6). Both fertilizer treatments resulted in Mn concentrations above a critical minimum limit of 50 mg kg^−1^ (Adeoye and Agboola 1985). Ear leaf concentrations of Zn and B are not influenced by macronutrients application, but are influenced by further addition of secondary and micronutrients. The concentration of B in maize ear leaves quadrupled when secondary and micronutrients were added to NPK fertilizer although no evidence was yet observed in improvements in the overall productivity. With the application of secondary and micronutrients, the Zn ear leaf concentrations are on average within the minimum critical limit of 15 mg kg^−1^ (Singh et al. 2005) and of 16 mg kg^−1^ (Welch and Graham 2000) although all treatments result in the Zn concentration above the critical limit of 10 mg kg^−1^ (Adeoye and Agboola 1985). Besides these micronutrient element concentrations in ear leaves, applying secondary and micronutrients increase sulfur (a secondary nutrient) concentrations in ear leaves relative to the NPK fertilizer treatment. The critical limits of most micronutrients for plant parts and also for soils are not yet determined for African conditions.

**Fig. 7:**
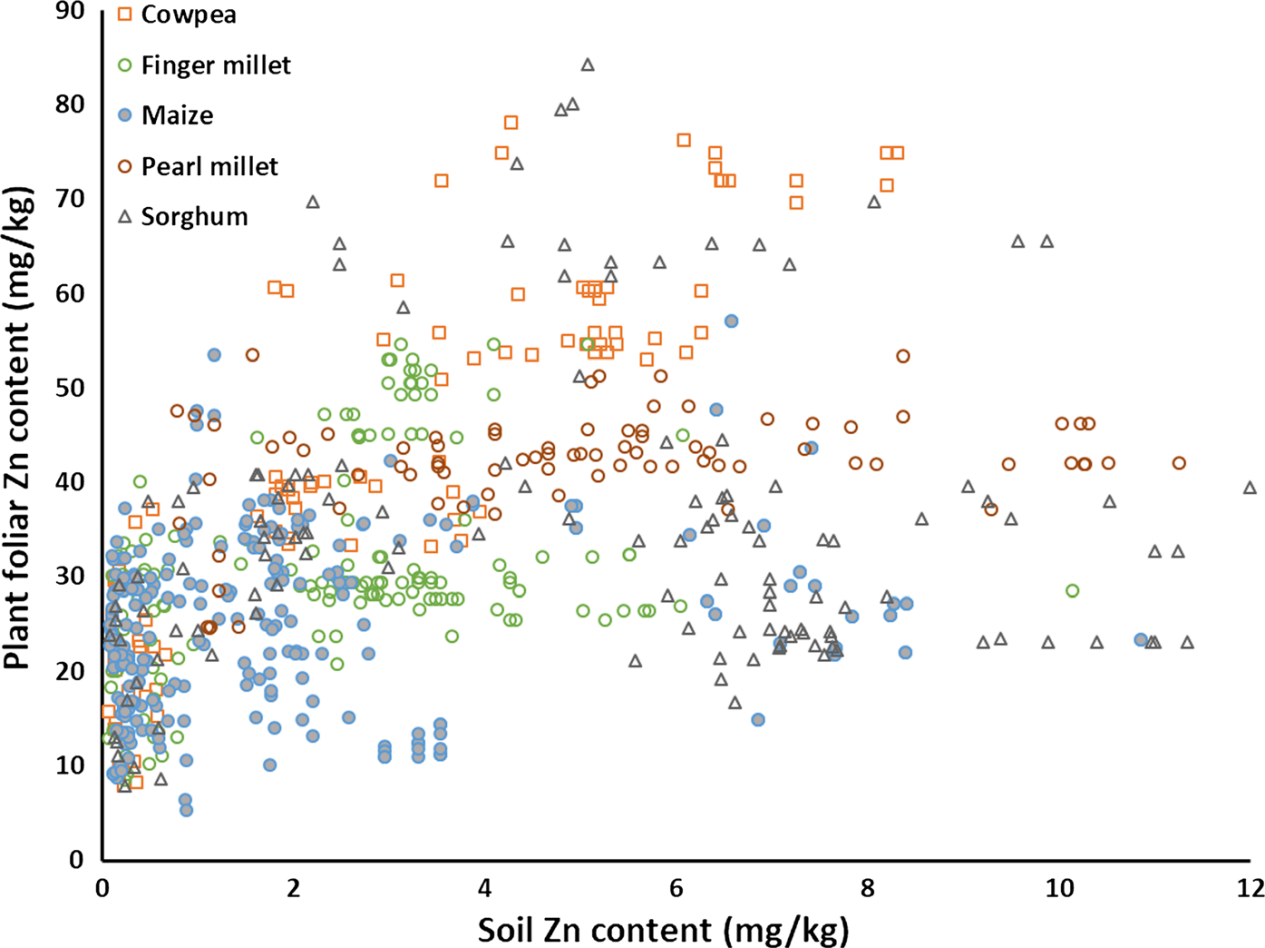
Concentrations of plant zinc for different crops at different soil Zn values for OFRA study locations across sub-Sahara Africa. All the samples are derived from treatments applied with N, P and K. The crops are maize (*Zea mays* L.), cowpea (*Vigna unguiculata* L.), pearl millet (*Pennisetum glaucum*), finger millet (*Eleusine coracana*) and sorghum (*Sorghum bicolor* L.)

As with maize grain yields, minimum plant foliar Zn concentrations increase with increasing soil Zn (Fig. 7). Also, the concentration of plant zinc increases with increasing soil Zn up to about 4–6 mg kg^−1^ of soil test Zn, especially for sorghum, cowpea and finger millet.

Evidence of changes in plant concentrations of micronutrients for SSA is based on experiments conducted mostly in individual farmer’s fields or concentrated in specific localities. To make region-wide nutritional changes using agronomic biofortification, major policy interventions are needed. For example, in Finland, a nationwide micronutrient study that involved enrichment of NPK fertilizers with 15 mg Se kg^−1^ prompted increased populace intake of selenium (above nutritional recommendations) by occasioning a 15-fold average increase in Se contents in the cereal grains (Alfthan et al. 2015).

### Effects of micronutrients on macronutrients use efficiency

Addition of micronutrients (S, Zn and B) in customized fertilizer blends (also with N, P and K) resulted in 50% increase in yields (+2.4 t ha^−1^) over commonly recommended NPS fertilizer (81N, 14P, 6S) pointing to increased utilization of N and P at higher rates where response curve would ordinarily level off (Van Vugt 2018). In a study conducted by One Acre Fund (unpublished), addition of customized blend, Mavuno fertilizer (containing NPK (10:26:10) plus secondary nutrients (Ca, Mg and S) and micronutrients (Zn, Cu, Mn, B and Mo) resulted in 75% increase (i.e., from 198 kg grain kg^−1^ P in control to 347 kg grain kg^−1^ P in the improved blend) in phosphorus use efficiency compared to local fertilizer recommendation. In India, annual application of NP plus 50% dose of S, B and Zn (i.e., full dose for the micronutrients were as follows: 30 kg ha^−1^ for S, 0.5 kg ha^−1^ B and 10 kg ha^−1^ Zn for maize and beans. N and P were applied as 100 kg N ha^−1^ and 26 kg P ha^−1^ for maize and 30 kg N and 26 kg P ha^−1^ for soybean) increased nitrogen use efficiency in maize by 36.4% relative to the NP control in maize and 21.7% in soybean, and also increased phosphorus use efficiency by 36.9% in maize and 19.4% in soybean (Chander et al. 2015). In Egypt, Khafagy et al. (2017) observed that including zinc fertilization of 20 and 40 kg Zn ha^−1^ increased rice grain N uptake by 17% and 28%, respectively, and similarly improved uptakes of P and K relative to the control treatment applied with N but not zinc. This is in line with El-Dahshouri (2018) who found that application of zinc increased macronutrient concentrations in wheat cultivars. In addition, application of zinc resulted to 17% increase in nitrogen use efficiency (i.e., from 43 kg grain kg^−1^ N to 50.3 kg grain kg^−1^ N) when nitrogen was applied during chickpea seed-filling stage, and 20% increase in nitrogen use efficiency (i.e., from 40.7 to 48.7 kg grain kg^−1^ N) when nitrogen was applied during the flowering stage (El-Habbasha et al. 2015), also in Egypt. Addition of NPK+ micronutrients increased nitrogen use efficiency and its apparent recovery for wheat by 39% and 36%, respectively, compared to application of only NPK (Malakouti 2008).

### Profitability of micronutrient fertilization

Crop response and profitability of micronutrient fertilization can often be underestimated. In some cases, increase in crop quality can be realized without increase in productivity, and vice versa. For example, despite the increase in maize grain quality following Se application, no significant increase in grain and stover yields was realized in Malawi (Chilimba et al. 2012). De Valença et al. (2017) confirmed that the application of Se-enriched fertilizers had potential to enhance Se concentrations in both maize and wheat grains, but not the yields. The fact that Se did not affect yields is probably due to the fact that this element is not essential for crop plants. On the other hand, the application of Zn fertilizer can improve not only productivity but grain Zn concentration by up to three to fourfold (Cakmak 2008). These quality aspects are not taken into account in our profitability assessment.

Most studies in SSA have applied micronutrients to crops as basal at planting. This application method often demands high application rates (higher costs) due to reduced recovery efficiency of applied micronutrients than with foliar application. On the other hand, soil application can result in positive residual effects on crop yield and quality which are commonly not assessed (an underestimation of economic benefits).

**Fig. 8:**
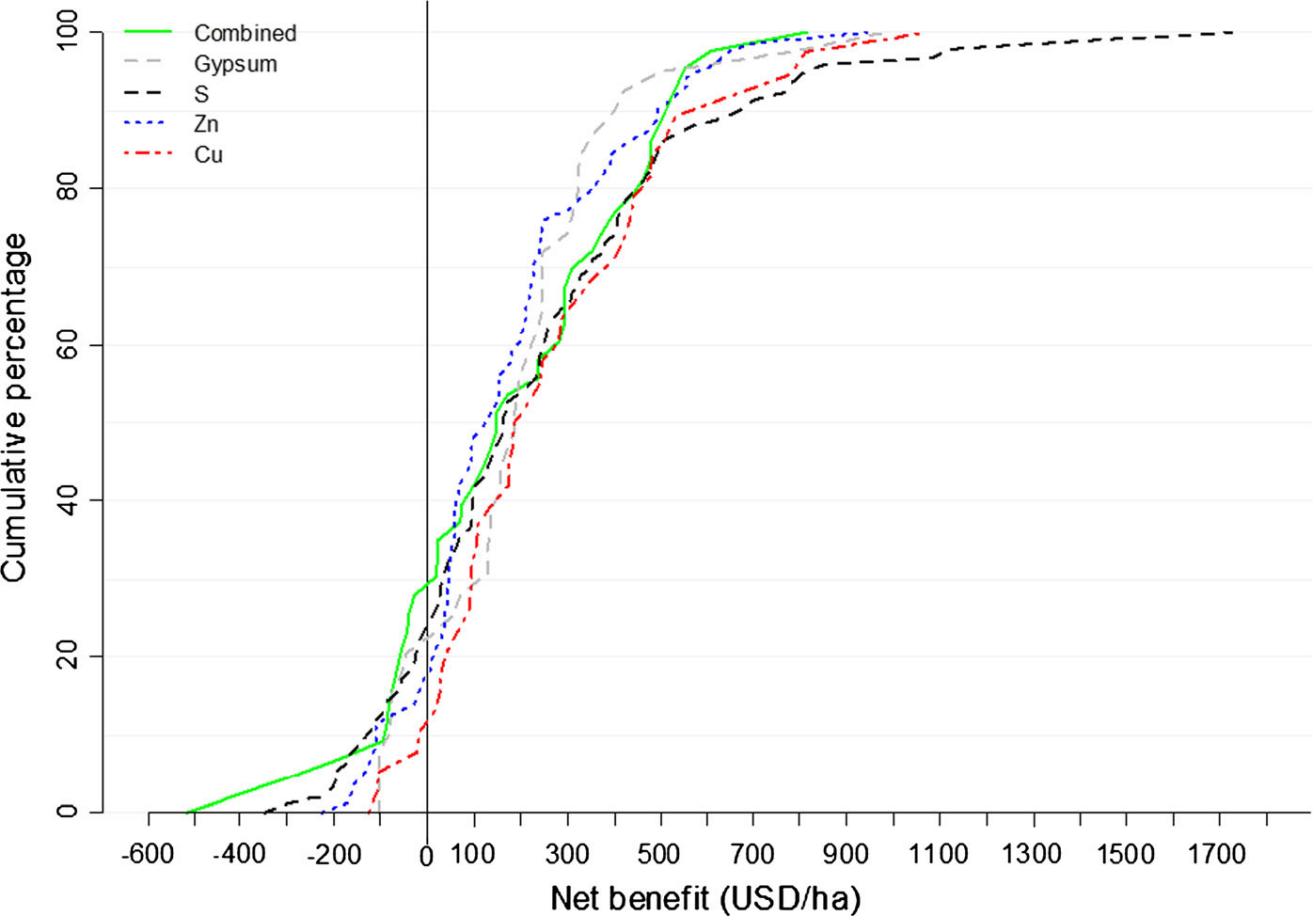
Distributions of net benefits and the associated cumulative percentages for combined secondary and micronutrients (combined), S, Zn and Cu as observed in SSA for maize. *N* = 44 for combined, 95 for S, 72 for Zn, 39 for Cu and 44 for gypsum. Black vertical line indicates zero benefit value when no benefits or losses are incurred. Few points where maize yield was > 10 t ha^−1^ were considered as erroneous and therefore omitted as this is not common in the region

**Fig. 9:**
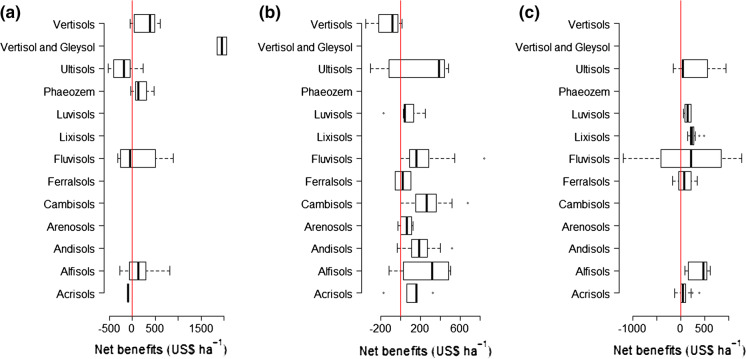
Range of net profits observed with **a** combined secondary and micronutrients, **b** S and **c** Zn as observed in SSA. Red lines indicate zero benefit value when no benefits or losses are incurred. Data used are for all crops

Application of secondary and micronutrients to maize has positive net benefits for 70%, 85%, 80% and 75% of the cases for combined secondary and micronutrients (i.e., combined), Cu, Zn and S, respectively (Fig. 8). Application of gypsum to maize (as a source of S but also contains Ca) resulted in positive net benefits in 80% of the cases. Positive net benefits of S in wheat production (*n* = 36), reaching a maximum of US$ 700, were observed in 94% of the cases (data not shown). High profitability is realized when low amounts of secondary and micronutrients are applied and/or resulting crop yield improvements are high. Unlike for combined secondary and micronutrients and Zn applications, S application is profitable across all soil types except in the fertile vertisols (Fig. 9).

### Factors affecting crop response to micronutrient applications

Factors such as source of the secondary and micronutrient and the mode of application such as through soil or foliar and the timing of application to crop development stages are discussed in the previous sections. A few more factors are presented here.

#### Crop species and cultivars

Variations in crop response to micronutrient applications may exist not only with different crops, but even with different cultivars of the same crop. In Egypt, zinc concentrations in wheat grains were found to vary with different wheat varieties planted (El-Habbasha et al. 2015). Besides wheat, variations in grain Zn concentrations in different rice germplasms have been reported (Welch and Graham 2002).

#### Soil properties

The plant available micronutrient content in the soil often shows considerable spatial variation (Hengl et al. 2017). This is probably the cause of at least part of the observed variation in crop response to micronutrient fertilization (see, for example, Figs. 2, 5). Where a nutrient element is severely deficient in the soil, the effect of fertilization is likely to be larger compared to when the soil nutrient supply is moderate or sufficient. Soil laboratories often use critical levels of plant available element concentrations in the soil, below which application of fertilizer containing the element is recommended. Soil nutrient element interactions can reduce efficiency and profitability of specific micronutrients. For example, elevated availability of soil P affects zinc availability to plants by precipitating the zinc, occasioning deficiency, and soil pH is important for the availability of most nutrient elements to crop roots.

### Effect on soil microbiota and environmental impacts

In SSA, there are no studies focused on direct effects of different micronutrient concentrations on soil microbial parameters and enzyme activities or on long-term environmental impacts. Nevertheless, there is evidence from elsewhere that when applied in the right dosage, micronutrient elements benefit soil biodiversity including soil microbial colonization, growth, mycorrhizal development, symbiotic nitrogen fixation and nodulation of legumes (Pollard et al. 1977; Alam et al. 2015; Farooq et al. 2018). The application of moderate amounts of boron up to 3 kg ha^−1^ increases soil fungal and bacterial populations, and phosphatase and dehydrogenase enzyme activities by between 18 and 34% during different growth periods relative to no application (Bilen et al. 2011). Application of 0.5 mg kg^−1^ of Mo increased nitrogenase enzyme activity (71%) and root nodule number (63%; Alam et al. 2015), while application of moderate Zn (15 kg Zn ha^−1^) increased nodule indices of cowpea by at least 38% (Upadhyay and Singh 2016). At high concentrations, for example, of boron (application of 9 kg B ha^−1^), the microbial growth and enzyme activities decrease due to impaired functions of cell membrane, and soil microbial structure is altered (Nable et al. 1997; Nelson and Mele 2007; Bilen et al. 2011; Vera et al. 2019). Similarly, high amounts of zinc decrease microbial biomass (by 41%; Chander and Brookes 1993) and reduce microbial species richness (by 38.5%; Moffett et al. 2003).

Long-term use of some chemical sources for restoring micronutrient deficiencies, for instance, zinc chelate-EDTA, may pose environmental challenges due to their characteristic low biodegradation and increased environmental persistence (Egli 2001; Meers et al. 2005). Prolonged application of ammonium sulfate fertilizers as a source of sulfur can alter soil pH and prompt ammonium accumulation to levels inhibitory to microbial communities, reducing soil microbial biomass and abundance (Geisseler and Scow 2014).

### Decision support for micronutrient application at multiple scales

#### Site-specific micronutrient fertilization

The core principle of precision crop production is to adapt crop management, including micronutrient fertilization to site-specific growth conditions (Gebbers and Adamchuk 2010). Consequently, nutrient use efficiency and profitability can be improved, and crop nutritional quality goals can be achieved to a larger degree, compared to if uniform rates were applied over large areas. The latter likely means unnecessary application (and poor profit) in some areas and at the same time insufficient application in other areas (*the Guldilock problem*; Foley et al. 2011). The core principles (and the expected benefits) apply at multiple spatial scales even if the specific term *precision agriculture* often refers to variable rate application within individual fields.

#### Decision support at multiple scales

Decisions on micronutrient fertilization are made or guided, directly or indirectly, at multiple spatial scales; national and sub-national authorities may decide on subsidies and for different inputs to crop production and may legislate on rates and types of fertilizers and lime products to important crops. Fertilizer companies may target their selling of specific fertilizer blends to regional needs and thus control what compounds are available in different regions, private and governmental extension service providers may provide advice on micronutrient application and individual farmers make the final decision to apply the fertilizer. At all these levels, decision support is needed and there are several options available. Recently, digital soil maps of several secondary and micronutrient elements (Ca, Mg, S, Fe, Mn, Zn, Cu, B) were published, covering the SSA (Hengl et al. 2015), providing spatial information on risks for micronutrient deficiencies. These can be further improved for adequate use in smaller regions by local adaptation (see principles by Söderström et al. 2017). There is also an option to collect new data by direct (soil or crop sampling + laboratory analysis) or indirect (proximal sensor measurements of soil or crop) measurements to diagnose micronutrient deficiencies at point locations (Nyambura et al. 2015; Towett et al. 2016; Piikki et al. 2016). It is, however, not enough that the decision support (the maps and the methods) exists. These need to be provided in a form that suits the different stakeholders, where also tailored decisions on fertilizations are made. New decision support systems are needed to bridge the gap between data and decisions.

The data presented on plant quality and profitability enhancement in this study and the productivity improvements shown earlier (Kihara et al. 2017) are strong arguments in favor of secondary and micronutrient fertilization. However, when linking soils to human malnutrition in SSA, Berkhout et al. (2019) concluded that although there is a significant positive link between soil micronutrient contents and malnutrition, agronomic biofortification is cost-ineffective. This claim will need to be further explored in the future to ascertain the bioavailability and uptake of the micronutrient-rich food and feed produced under agronomic biofortification.

## Conclusion

The synthesis of scientific data and literature shows that:

- There is widespread but variable micronutrient deficiencies in arable soils in SSA, and more than one micronutrient elements are often deficient at the same geographic location.
- Application of Zn and Se increased micronutrient concentrations in harvested cereal and legume grains, but the concentrations varied considerably as the dataset used included multiple cultivars and sites.
- It was profitable to apply fertilizers containing micronutrient elements. Application of S was profitable in almost all cases, while the profitability of Zn application was more variable. Profitability also varied with soil type.
- There is a general lack of public information on how application of other nutrients than N, P and K affects crop yield and nutritional quality in SSA. Most public information available is on the effects of Zn application.
- To raise the nutritional quality of major food crops in SSA while striving toward a resource use efficient and profitable crop production, fertilizer sources/types and rates need to be tailored to local soil and cropping conditions, crop and cultivar type (i.e., need for customized blends).
- Transforming the current food systems to take into account human nutritional requirements, especially through agronomic approaches, is urgently needed.
- Although the call for more work to link fertilizer technology and improvement of the nutritional quality of staple food crops that feed the world’s malnourished poor has been made since 2012, there is still a dearth of knowledge on this, especially in SSA.
